# Response of ecosystem CO_2_ fluxes to grazing intensities – a five-year experiment in the Hulunber meadow steppe of China

**DOI:** 10.1038/s41598-017-09855-1

**Published:** 2017-08-25

**Authors:** R. R. Yan, H. J. Tang, S. H. Lv, D. Y. Jin, X. P. Xin, B. R. Chen, B. H. Zhang, Y. C. Yan, X. Wang, Philip J. Murray, G. X. Yang, L. J. Xu, L. H. Li, S. Zhao

**Affiliations:** 1 Institute of Agricultural Resources and Regional Planning, Chinese Academy of Agricultural Sciences, Beijing, 100081 P.R. China; 2 College of Science Inner Mongolia Agricultural University, , Hohhot, Inner Mongolia, 010018 China; 3 Rothamsted Research, North Wyke, Okehampton, Devon, EX20 2SB UK; 4 State Key Laboratory of Vegetation and Environmental Change, Institute of Botany, Chinese Academy of Sciences, Beijing, P.R. China; 5 Inner Mongolia Agricultural University, Hohhot, Inner Mongolia, 010070 China

## Abstract

Grazing is the primary land use in the Hulunber meadow steppe. However, the quantitative effects of grazing on ecosystem carbon dioxide (CO_2_) fluxes in this zone remain unclear. A controlled experiment was conducted from 2010 to 2014 to study the effects of six stocking rates on CO_2_ flux, and the results showed that there were significant differences in CO_2_ fluxes by year, treatment, and month. The effects of light and intermediate grazing remained relatively constant with grazing year, whereas the effects of heavy grazing increased substantially with grazing duration. CO_2_ flux significantly decreased with increasing grazing intensity and duration, and it was significantly positively correlated with rainfall, soil moisture (SM), the carbon to nitrogen ratio (C/N ratio), soil available phosphorus (SAP), soil NH_4_^+^-N, soil NO_3_^−^N, aboveground biomass (AGB), coverage, height, and litter and negatively correlated with air temperature, total soil N (TN) and microbial biomass N (MBN). A correspondence analysis showed that the main factors influencing changes in CO_2_ emissions under grazing were AGB, height, coverage, SM, NH_4_^+^-N and NO_3_^−^N. Increased rainfall and reduced grazing resulted in greater CO_2_ emissions. Our study provides important information to improve our understanding of the role of livestock grazing in GHG emissions.

## Introduction

The flux of carbon dioxide (CO_2_) plays a critical role in the carbon (C) cycle of terrestrial ecosystems and is an important index of soil bioactivity, fertility and ventilation^1–3^. The production of soil CO_2_ primarily depends on the mineralization of soil organic matter, which involves microorganisms, and the respiration of soil animals and plants. The production of CO_2_ is the result of multiple factors, including bio-metabolic and biochemical processes. Many factors that contribute to soil biological processes and biochemical reaction velocities can affect the rate of CO_2_ emissions^4^. Hui *et al*.^5^ showed that fluctuations in CO_2_ fluxes are mainly caused by climatic variations via direct effects on the physiological processes of photosynthesis and respiration and via indirect effects on biological and ecological processes that regulate C uptake and loss^5^.

Among terrestrial ecosystems, grasslands are one of the most important biome types, and they play an important role in regulating the global C cycle, as they comprise approximately 40% of the global land area^6^. Several studies have shown that temperate grasslands can act as both sinks and sources of CO_2_^7–9^. Other studies have also simultaneously measured diurnal, seasonal and annual variations of ecosystem CO_2_ exchange on the Tibetan plateau^10^. Differences and changes in land management can be expected to affect the C sequestration rate of these ecosystems^11^, which in turn affects atmospheric CO_2_ concentrations^12, 13^. Grazing is the most common land use practiced in grassland ecosystems^14^. Grazing animals affect organic matter quantity and quality via several mechanisms, including the return of animal wastes to the soil, alteration of plant productivity and vegetation composition (which govern the quality and amount of plant-leaf–root litter exudates entering the soil), and changes in the activity and composition of soil microbial communities. These changes subsequently affect the rates of nutrient cycling, creating feedback loops in plant productivity that affect ecosystem CO_2_ fluxes^15^. Furthermore, grazing also affects the rates of soil C cycling processes that are direct or indirect sources of CO_2_^16^. Therefore, understanding the relationships between grazing management and C cycling within the plant–animal–soil continuum and its many feedback loops and interactions is critical for the development of efficient and effective CO_2_ mitigation strategies for livestock grazing systems.

Several studies^17, 18^ have shown that grazing can alter C emissions from soils to the atmosphere. However, research on the impact of human-related activities on the source and sink functions of the main greenhouse gases has consistently found that grazing does not change the properties of the soil as a source of CO_2_. Researchers have found that grazing decreases CO_2_ emissions^19^, while others have found that grazing increases CO_2_ emissions^20^ or has no effect^21^. Such discrepancies suggest that the response of CO_2_ emissions to grazing may vary with grazing intensity, grazing history, climate and soil type^20, 22^.

The Hulunber grasslands in Inner Mongolia cover an area of approximately 9.97 × 10^6^ km^2^ and are located in the eastern part of the Eurasian grassland region. These grassland ecosystems are important, typical native grasslands dominated by the grass *Leymus chinensis* that are essential for livestock farming in northern China. However, steppe ecosystems in China are suffering from increased stocking rates resulting from the sharp increase in the demand for animal products^23^. Most of the area is now degraded, which has resulted in serious constraints on livestock management^24^ and considerable effects on CO_2_ fluxes^25^. However, current knowledge cannot explain the mechanisms responsible for these grazing effects. Furthermore, the influence of quantified grazing intensity on CO_2_ flux has yet to be carefully studied. Quantifying CO_2_ emissions and the uptake of different grazing stocking rates is therefore an essential step for understanding the roles of semi-arid temperate grasslands in a context of global climate change.

In this study, the impacts of different cattle grazing intensities on ecosystem CO_2_ fluxes were examined during the growing seasons from 2010 to 2014 in the Inner Mongolian meadow steppe of China. Relevant environmental factors and plant and soil properties were observed concurrently. In this paper, we test the hypothesis that grazing intensity causes changes in ecosystem CO_2_ fluxes during the growing season by (1) establishing the mechanisms underlying any changes through the examination of the relationships between ecosystem CO_2_ fluxes and environmental, soil and biological factors and (2) exploring the interactions between the grazing intensities and plant community and soil factors using the correspondence analysis method.

## Results

### Monthly and yearly variations in CO_2_ fluxes due to grazing intensity

**Figure 1:**
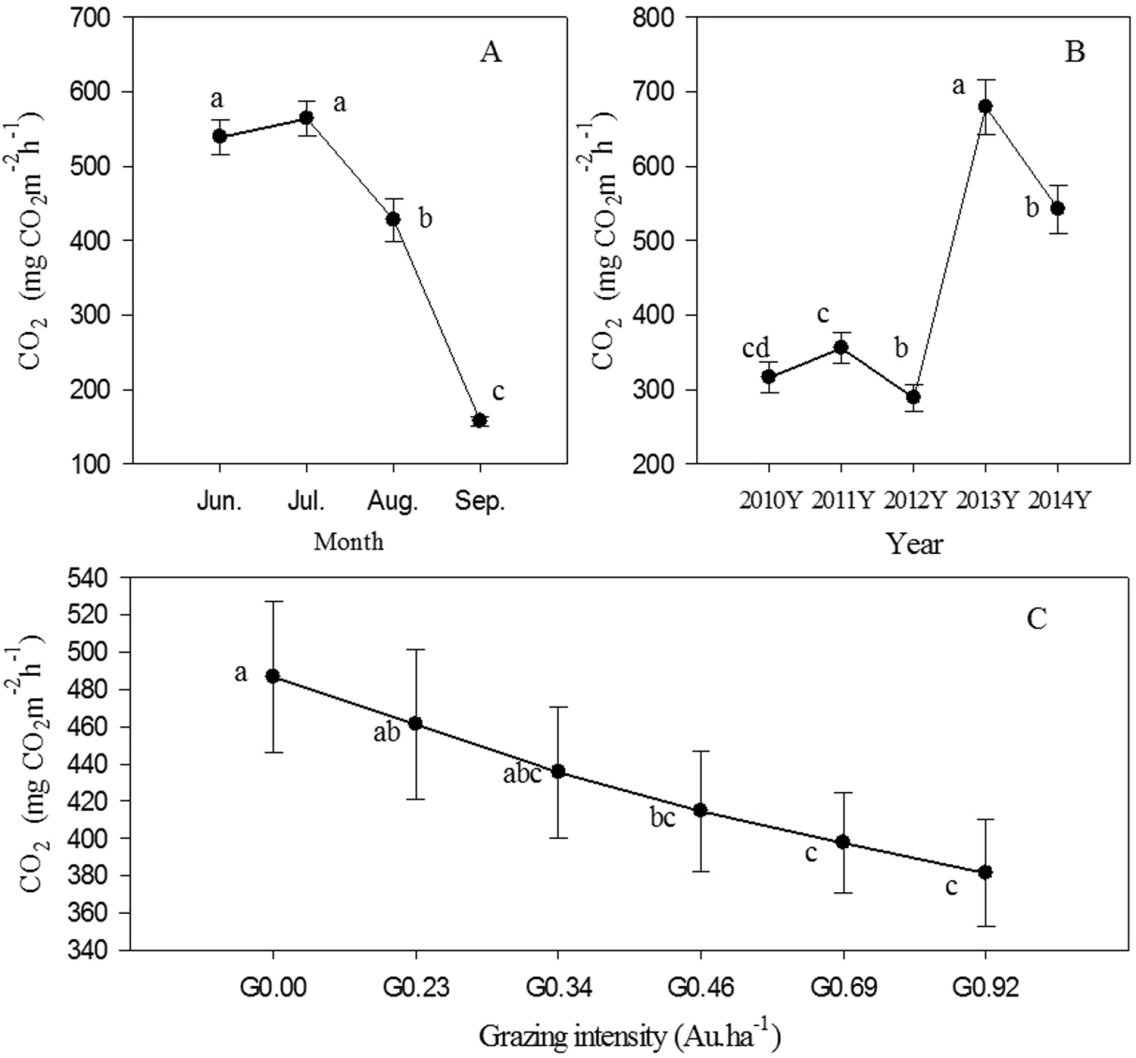
(**A–C)**. Changes in ecosystem CO_2_ fluxes (mean ± s.e.) with respect to the month, year and grazing intensity during the growing and grazing period in 2010, 2011, 2012, 2013 and 2014. The bars represent the means of three replicate plots (±s.e.). Different letters indicate significant differences among the CO_2_ fluxes in different months, years and grazing intensities.

We examined how the CO_2_ fluxes were affected by grazing intensity and grazing duration. Mean ecosystem CO_2_ emissions from 2010 to 2014 exhibited significant variance (*p* < 0.05) between years, seasons and treatments (Table 1). Multiple comparison tests were conducted to evaluate the differences in CO_2_ emissions under different grazing intensities and different monthly and yearly growing season variations. The results show that the mean ecosystem CO_2_ flux of the Hulunber steppe was positive and was thus a source of C during the growing and grazing season. The peak CO_2_ fluxes typically occurred after effective rainfall, so the mean CO_2_ emissions rate exhibited significant temporal variations during the growing season. These emission rates were greater in July (576 mg CO_2_ m^−2^ h^−1^) and June (539 mg CO_2_ m^−2^ h^−1^) than in September (159 mg CO_2_ m^−2^ h^−1^) (Fig. 1A). Furthermore, the CO_2_ flux differed significantly among years (Fig. 1B). The mean CO_2_ emission rates over the growing season in the wetter years of 2013 (625 mg CO_2_ m^−2^ h^−1^) and ^2^014 (540 mg CO_2_ m^−2^ h^−1^) were significantly higher than those in the average precipitation year, 2010 (316 mg CO_2_ m^−2^ h^−1^), and the dry years of 2011 (356 mg CO_2_ m^−2^ h^−1^) and ^2^012 (289 mg CO_2_ m^−2^ h^−1^) for all treatments (*p* < 0.05) (Fig. 1B). These inter-year variations may have been the result of differences in the climate conditions; the amounts of rainfall in 2010, 2011 and 2012 were much lower than in 2013 and 2014. Therefore, regardless of the grazing treatment, the CO_2_ flux from the soil changes significantly in response to variations in temperature and rainfall.

**Table 1:** Repeated-measures ANOVA of degrees of freedom (df), sum of squares, mean square, F values, and probabilities (Pr > F) of the CO_2_ fluxes for the effects of year, treatment, and month under different grazing intensities.

Source of variation	df	Sum of squares	Mean square	F value	Pr > F
Model	12	15,556524.22	1296377.02	51.06	<.0001
Years	4	64,93533.51	1623383.38	63.94	<.0001
Treatments	5	47,9499.27	95899.86	3.78	0.0024
Months	3	75,19700.48	2506566.83	98.73	<.0001
Error	336	85,30732.98	25389.09		
Total variation	348	24,087257.20			

Data from five experimental years were used for the statistical analysis. Different treatments were analysed separately. There were three replicates for each grazing intensity each year for the CO_2_ flux data.

During the 5 years of grazing, the CO_2_ emissions rate changed greatly among the different grazing treatments (Fig. 1C). Over time, the CO_2_ flux of the grasslands decreased significantly with increased grazing intensity. Relative to the control, the CO_2_ fluxes under different grazing intensities decreased by 7.9–23.8%, and the statistical analysis showed that the CO_2_ fluxes of the no grazing treatment G0.00 (495 mg CO_2_ m^−2^ h^−1^) and the light grazing treatment G0.23 (455 mg CO_2_ m^−2^ h^−1^) were significantly higher than the heavy grazing treatment G0.69 (388 mg CO_2_ m^−2^ h^−1^) and G0.92 (377 mg CO_2_ m^−2^ h^−1^) (*p* < 0.05). The CO_2_ fluxes under light (G0.34) and intermediate grazing treatments (G0.46) are between those of G0.00 and the heavy grazing treatments (Fig. 1C).

Monthly and yearly cumulative CO_2_ fluxes from June to October in 2010, 2011, 2012, 2013 and 2014 under different grazing intensities are shown in Table 2. The yearly cumulative CO_2_ fluxes in 2010 and 2011 did not differ significantly among the treatments (*p* > 0.05), but the CO_2_ flux was substantially influenced by grazing intensity from 2012 onwards. Significantly greater fluxes were released by the ungrazed treatment than by the G0.92 treatment in 2013 and 2014 (*p* < 0.05). Relative to the CO_2_ flux of the ungrazed treatment, the CO_2_ fluxes associated with the different grazing intensities decreased by 6.4–29.1% in 2012, 7.2–32.5% in 2013 and 9.1–32.2% in 2014. Thus, despite the variance in annual rainfall and temperature, the grazing treatment leads to significant changes in the CO_2_ fluxes. The influence of light and intermediate grazing remained relatively constant with the duration of grazing, whereas the influence of heavy grazing increased substantially.

**Table 2:** Monthly and yearly cumulative fluxes of ecosystem CO_2_ (kg CO_2_ ha^−1^) from June to October in 2010, 2011, 2012, 2013 and 2014 under different grazing intensities. The bars represent the means of three replicate plots (±s.e.). Different letters indicate significant differences among the levels of grazing intensity at both monthly and yearly scales over the growing season (one-way ANOVA, P < 0.05).

Year	Time	G0.00	G0.23	G0.34	G0.46	G0.69	G0.92
2010	Jun.	1,745.16 ± 263.29a	1,785.67 ± 150.57a	1,689.17 ± 153.44a	1,758.04 ± 165.00a	1,408.86 ± 15.13a	1,618.08 ± 141.28a
	Jul.	1,155.03 ± 292.92a	1,329.22 ± 292.84a	1,997.80 ± 428.81a	1,729.52 ± 425.75a	1,414.91 ± 406.22a	1,494.64 ± 162.06a
	Aug.	660.75 ± 9.14a	761.41 ± 167.46a	874.63 ± 165.27a	595.71 ± 48.66a	1,461.06 ± 273.08a	1,227.79 ± 561.81a
	Sep.	569.74 ± 43.15a	330.77 ± 44.93b	396.35 ± 24.39ab	361.43 ± 22.83b	501.41 ± 71.30ab	433.52 ± 96.01ab
	Yearly	4,130.69 ± 439.44a	4,207.06 ± 106.08a	4,957.94 ± 440.32a	4,444.69 ± 638.33a	4,786.25 ± 682.61a	4,774.02 ± 719.15a
2011	Jun.	963.36 ± 137.78a	981.06 ± 152.88a	947.27 ± 142.38a	924.23 ± 232.75a	1,003.13 ± 164.09a	879.10 ± 80.85a
	Jul.	2,078.23 ± 60.82ab	2,264.30 ± 264.98a	2,052.03 ± 200.57ab	1,775.53 ± 124.03ab	1,973.25 ± 149.13ab	1,596.15 ± 128.06b
	Aug.	1,935.30 ± 302.89a	1,734.47 ± 194.17a	1,735.69 ± 102.57a	1,602.90 ± 252.05a	1,626.32 ± 201.03a	1,480.47 ± 24.50a
	Sep.	659.43 ± 110.73a	382.96 ± 48.64b	523.75 ± 48.72ab	523.72 ± 33.09ab	586.48 ± 84.75ab	504.22 ± 44.76ab
	Yearly	5,636.31 ± 542.01a	5,362.79 ± 656.10a	5,258.74 ± 97.81a	4,826.38 ± 580.67a	5,189.18 ± 275.70a	4,459.94 ± 97.14a
2012	Jun.	2,242.23 ± 114.30a	1,426.69 ± 167.97b	1,870.54 ± 83.40a	1,882.96 ± 152.83a	1,319.24 ± 95.40b	1,390.30 ± 60.59b
	Jul.	1,552.83 ± 334.35a	1,469.29 ± 30.07a	1,372.04 ± 77.49a	1,395.91 ± 127.16a	1,235.01 ± 196.38a	1,230.67 ± 64.55a
	Aug.	595.86 ± 180.22a	542.31 ± 26.54a	583.46 ± 209.03a	567.05 ± 82.35a	395.68 ± 16.28a	481.69 ± 75.46a
	Sep.	489.23 ± 149.78a	510.86 ± 89.51a	534.90 ± 146.72a	724.56 ± 89.65a	511.85 ± 32.66	612.56 ± 12.30a
	Yearly	4,880.15 ± 769.35a	3,949.14 ± 283.93ab	4,360.93 ± 449.70ab	4,570.48 ± 360.79ab	3,461.78 ± 228.00b	3,715.22 ± 41.38b
2013	Jun.	3,004.08 ± 348.97ab	3,205.02 ± 116.51a	2,644.32 ± 213.22abc	2,009.08 ± 209.98c	2,376.25 ± 170.48bc	2,548.32 ± 190.41abc
	Jul.	4,092.64 ± 1,299.52a	3,508.45 ± 225.02a	3,300.56 ± 507.56a	2,496.08 ± 367.49a	2,618.93 ± 346.78a	2,440.88 ± 95.95a
	Aug.	3,398.43 ± 223.95a	3,234.77 ± 338.44a	2,565.50 ± 685.90a	3,481.97 ± 368.84a	2,209.72 ± 143.04a	2,134.63 ± 390.83a
	Sep.	654.55 ± 0.00a	399.84 ± 2.25a	486.18 ± 89.47a	373.11 ± 0.00a	420.86 ± 0.00a	401.65 ± 20.63a
	Yearly	11,149.71 ± 1653.54a	10,348.07 ± 100.59ab	8,996.56 ± 1425.75ab	8,360.23 ± 310.02ab	7,625.76 ± 260.22b	7,525.48 ± 498.91b
2014	Jun.	3,569.61 ± 328.70a	3,296.09 ± 48.76ab	2,778.61 ± 402.82abc	2,498.79 ± 205.06bc	2,063.21 ± 420.55c	2,387.11 ± 253.24bc
	Jul.	2,825.30 ± 232.83a	2,958.50 ± 551.89a	2,438.20 ± 429.00a	2,215.07 ± 64.19a	2,072.67 ± 24.46a	2,201.58 ± 726.82a
	Aug.	2,311.51 ± 422.89a	1,750.93 ± 543.67a	1,491.49 ± 402.19a	1,492.64 ± 285.68a	1,901.53 ± 237.58a	1,374.54 ± 521.53a
	Sep.	1,121.46 ± 109.39a	925.82 ± 84.67ab	717.61 ± 7.64b	696.19 ± 153.44b	852.33 ± 115.36ab	695.37 ± 170.61b
	Yearly	9,827.87 ± 783.37a	8,931.33 ± 1222.98ab	7,425.91 ± 1191.78bc	6,902.68 ± 304.68bc	6,889.74 ± 409.62c	6,658.60 ± 1654.84c

### Analysis of changes in CO_2_ fluxes

**Figure 2:**
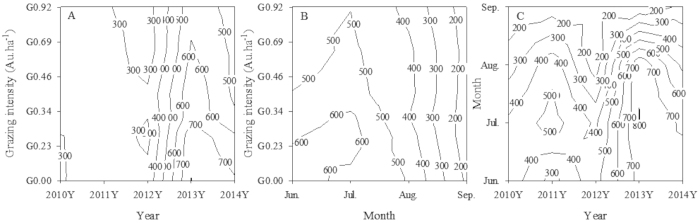
(**A–C)**. Pairwise contour map analysis of changes in mean ecosystem CO_2_ fluxes (mg CO_2_ m^−2^ h^−1^) from June to September for five years.

To visualize the relationships of the three variables (year, month and grazing treatment) to the CO_2_ fluxes, we used a pairwise analysis, and the results showed that, during the growing season, the mean yearly CO_2_ emissions rate of the grassland did not change between treatments from 2010 to 2012. However, after 4–5 years of grazing, i.e., in 2013 and 2014, the ecosystem CO_2_ emission rates from the grassland were lower under higher grazing pressures (Fig. 2A), and peak fluxes occurred under grazing pressures of 0.00–0.34. Therefore, increased rainfall and less grazing lead to higher CO_2_ fluxes, and ecosystem CO_2_ fluxes under light grazing are higher than those under heavy grazing.

Substantial temporal fluctuations in the CO_2_ flux occurred during the growing seasons among the different grazing intensities. The ecosystem CO_2_ emissions rate declined with increasing grazing intensity from June to August, whereas there was no change in the CO_2_ flux among the different grazing intensities after August. The highest CO_2_ emissions were associated with light grazing intensity (0.34 AU ha^−1^) from June to July (Fig. 2B). In addition, the yearly changes in the CO_2_ emissions rate throughout the grazing period showed a bimodal distribution. In contrast, the seasonal dynamics of the ecosystem CO_2_ fluxes exhibited a distribution that increased from mid-June to late July and decreased after August (Fig. 2C).

### Effect of the responses of the impact factors to grazing intensity on CO_2_ fluxes

**Figure 3:**
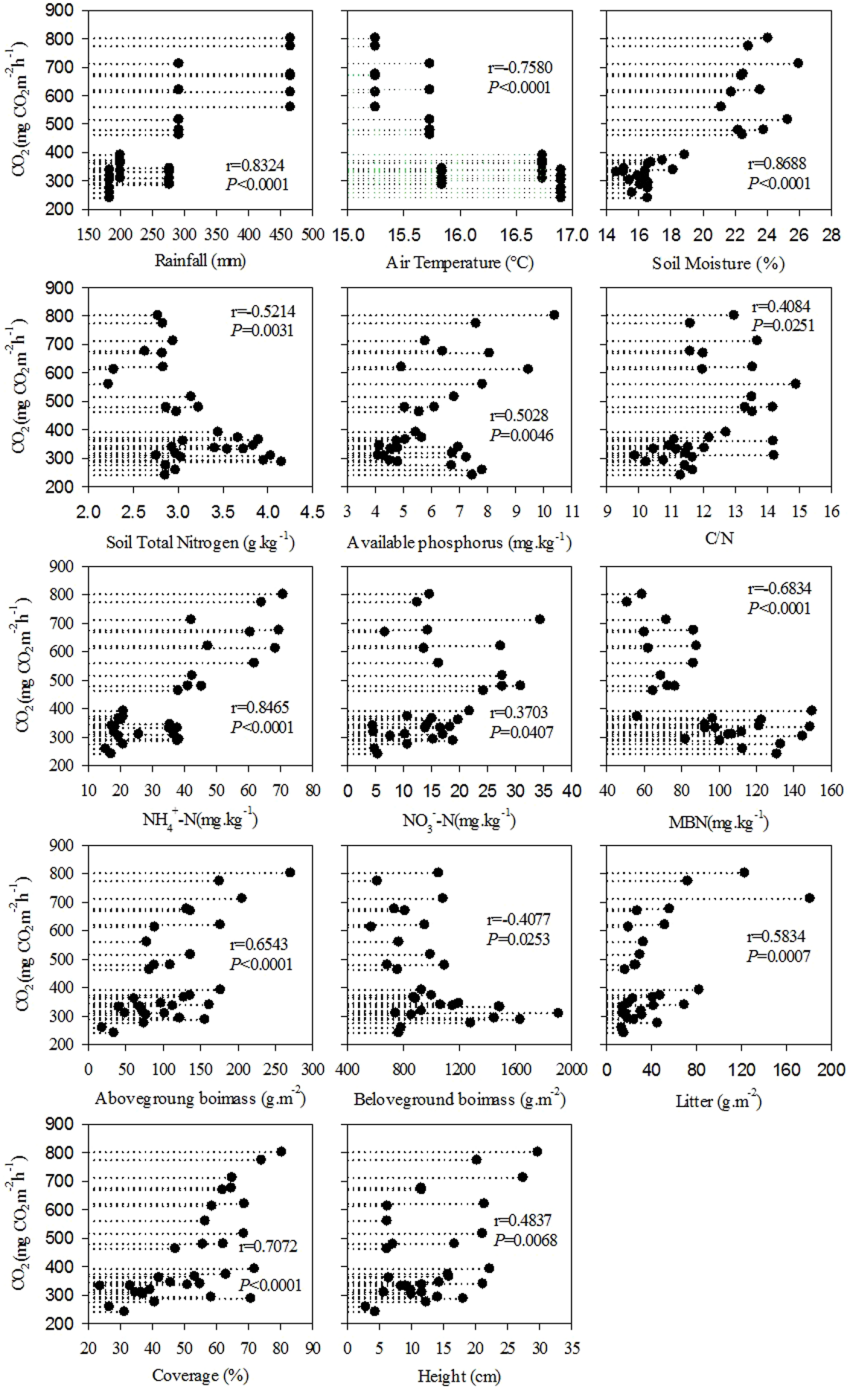
Relationships between the mean ecosystem CO_2_ fluxes and meteorological factors (rainfall and air temperature), soil factors (soil moisture, soil total nitrogen, C/N, soil available phosphorus, NH^4+^-N, NO^3^-N and microbial biomass nitrogen) and vegetation factors (aboveground biomass, belowground biomass, litter, coverage and height) from all plots across five years.

The relationships between ecosystem CO_2_ fluxes and impact factors (including climate, soil and vegetation factors) differed substantially. Across all treatments and years (Fig. 3), for climate factors, CO_2_ flux was shown to be significantly positively correlated with rainfall (r = 0.832, *p* < 0.001) but significantly negatively correlated with air temperature (r = −0.758, *p* < 0.001). There were significant positive correlations between ecosystem CO_2_ flux and soil moisture (SM) (r = 0.869, *p* < 0.001), the C to nitrogen ratio (C/N ratio) (r = 0.408, *p* < 0.05), soil available phosphorus (SAP) (r = 0.503, *p* < 0.01), soil ammonium N (NH_4_^+^-N) (r = 0.847, *p* < 0.001), soil nitrate N (NO_3_^—^N) (r = 0.350, *p* < 0.05), aboveground biomass (AGB) (r = 0.654, *p* < 0.001), plant cover (r = 0.707, *p* < 0.001), plant height (r = 0.484, *p* < 0.01) and litter quantity (r = 0.583, *p* < 0.001). In contrast, there were negative relationships between ecosystem CO_2_ flux and soil total N (TN) (r = 0.521, *p* < 0.01), soil microbial biomass N (MBN) (r = 0.683, *p* < 0.001) and belowground biomass (BGB) (r = 0.408, *p* < 0.05). No significant relationships were detected between ecosystem CO_2_ flux and soil pH, soil bulk density (SBD), soil organic C (SOC), soil total phosphorus (TP), soil total potash (TK), soil available N (AN), soil available potash (AK) and soil microbial biomass C (MBC) (Supplementary Fig. 1).

### Interactions between CO_2_ fluxes and grazed grassland ecosystem plant and soil factors

**Figure 4:**
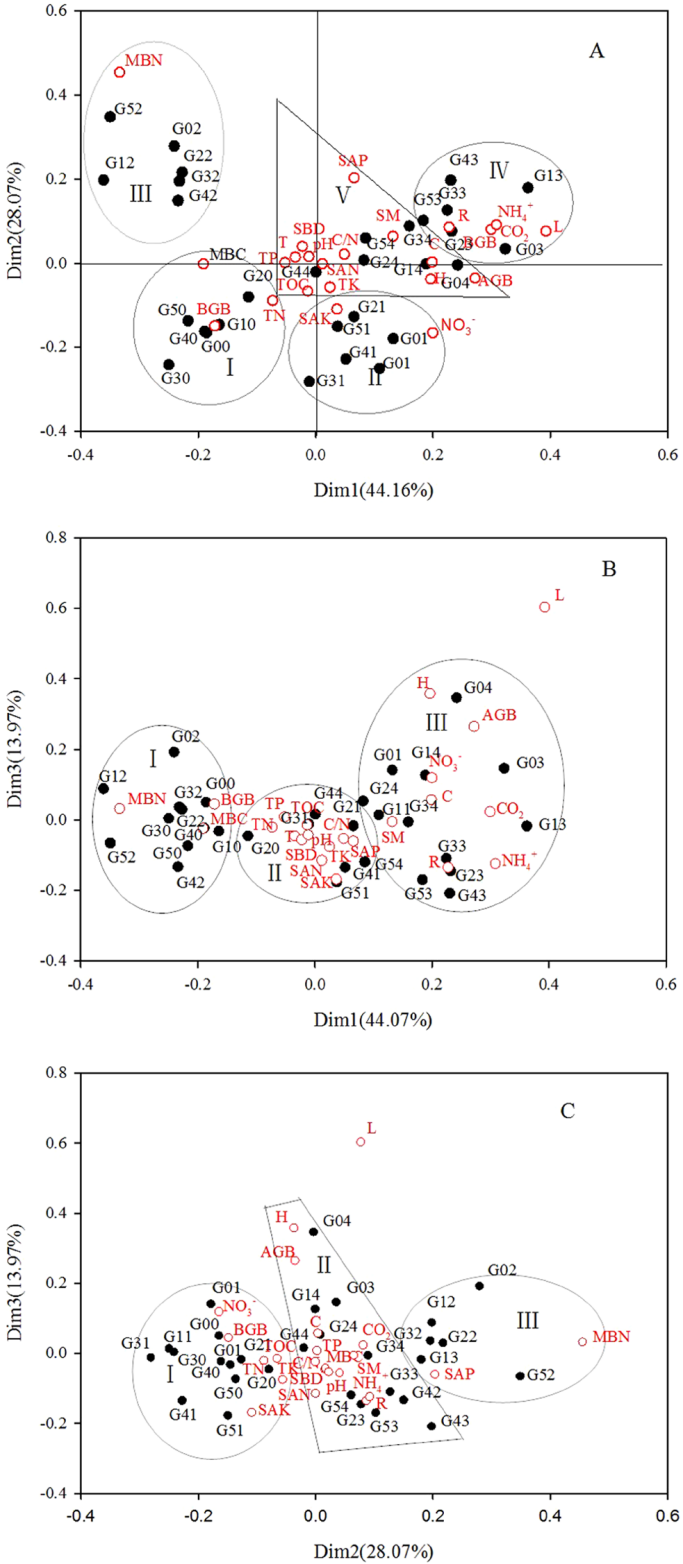
(**A–C)**. Correspondence analysis results between grazing intensity, ecosystem CO_2_ fluxes and environment factors. Dim1, Dim2 and Dim3 represent for the eigenvectors of different grazing intensities and different indicators. Figure 4A is a correspondence analytical figure of Dim 1 and Dim 2, and Fig. 4B is a correspondence analytical figure of Dim 1 and Dim 3. Figure 4C is a correspondence analytical figure of Dim 2 and Dim 3. In the figure, G00, G10, G20, G30, G40 and G50 refer to the 6 treatments in 2010; G01, G11, G21, G31, G41 and G51 refer to the treatments in 2011; G02, G12, G22, G32, G42, G52 refer to the treatments in 2012; G03, G13, G23, G33, G43, G53 refer to the treatments in 2013; and G04, G14, G24, G34, G44 and G54 refer to the treatments in 2014. The red circles denote the variables included in the diagram. CO_2_ represents the ecosystem CO_2_ fluxes. The climate factors are R (rainfall) and T (air temperature). The plant community variables are AGB (aboveground biomass), C (coverage), H (height), BGB (belowground biomass) and L (litter). The soil environment variables are SBD (soil bulk density), pH and SM (soil moisture). The soil nutrient variables are SOC (soil organic carbon), TN (total nitrogen), TP (total phosphorus), TK (total potassium), SAN (soil available nitrogen), SAP (soil available phosphorus), SAK (soil available potassium), C/N (carbon to nitrogen ratio), NH_4_^+^ (soil NH_4_^+^-N) and NO_3_^−^ (soil NO_3_^−^N).

Correspondence analysis (Fig. 4) was conducted using 25 indicators (including meteorological, soil and vegetation factors) for different years and different grazing treatments. The first axis (Dim 1) variance contribution rate was 44.2%, the second axis (Dim 2) variance contribution rate was 28.1%, the third axis (Dim 3) variance contribution rate was 14.0%, and the total variance contribution rate was 86.2% (>85%).

The results of the correspondence analysis between Dim 1 and Dim 2 are shown in Fig. 4A. Region I represents treatments G00–G50 in 2010, including MBN, BGB and TN, indicating relatively high values of MBN, BGB and TN. Region II represents treatments G01–G51 in 2011, including SAK and NO_3_^−^, indicating relatively high values of SAK and NO_3_^−^. Region III represents treatments G02–G52 in 2012, including MBC, indicating a relatively high value of MBC. Region IV represents G03-G53 in 2013, including rainfall, CO_2_, NH_4_^+^ and litter, indicating relatively high levels of rainfall, CO_2_ and NH_4_^+^ and low levels of litter. Other indicators are concentrated in Region V. Because each region includes various grazing treatment regions, with indicators scattered in each region, the differences in years affect the various indicators through annual differences in rainfall and temperature, which lead to variations in the plant community and soil microorganisms.

A correspondence analytical figure of Dim 1 and Dim 3 is shown in Fig. 4B. Three regions have been delineated. Region I includes treatments G00, G10, G30, G40, and G50 in 2010 and G02, G12, G22, G32, G42, and G5 in 2012, and the indicators BGB, MBN and MBC. Region II includes G20 in 2010, and G21, G31, G41, and G51 in 2011, G44 and G54 in 2014, and the indicators SOC, TN, TP, TK, C/N, SAN, SAP, SAK, temperature, SBD and pH. Region III includes G01 and G11 in 2011 and G03-G53 in 2013, G04, G24, and G34 in 2014, and the remaining indicators. The information shown in the three regions is related to the CO_2_ emissions. The CO_2_ emissions are relatively low in Region I, intermediate in region II, and high in region III. Thus, low soil CO_2_ emissions are closely related to MBN, MBC and BGB, whereas high ecosystem CO_2_ emissions are closely related to rainfall, AGB, height, coverage, SM, NH_4_^+^ and NO_3_^−^. The latter factors in general exhibit significant positive correlations with the CO_2_ fluxes, corroborating the findings of the correlation analysis. The indicators in region II are soil nutrition indicators that plot close to the origin of the coordinate axis. These soil indicator factors have an intermediate level of effect on CO_2_ emissions and are relatively stable and little influenced by other factors.

Figure 4C is a correspondence analytical figure of Dim 2 and Dim 3. Region I represents treatments G00–G50 in 2010 and G01–G51 in 2011 as well as the indicators SOC, TN, TK, SAK, NO_3_^−^ and litter. Region II represents treatments G42 in 2012, G03, G23, G33, G43, G53 in 2013, and G04–G54 in 2014 as well as the indicators TP, C/N, SAN, NH_4_^+^, MBN, AGB, height, coverage, CO_2_, SM, SBD, pH, rainfall and temperature. Region III represents treatments G02, G12, G22, G32, and G52 in 2012 and G13 in 2013 as well as the indicators SAP and MBC. Based on the average rainfall and temperature data from the observation period, Region I features years with low rainfall and high temperatures; Region II features years with high rainfall and low temperatures; and Region III features years with low rainfall and high temperatures. CO_2_ emissions tend to be higher in years with more rainfall and lower temperatures.

In general, the three corresponding analytical figures demonstrate that Dim 1 and Dim 2 reflect yearly variations in the CO_2_ flux and that Dim 1 and Dim 3 indicate CO_2_ flux variations and their relationships with the studied factors. Figure 4C shows the extent of the effects of annual rainfall and temperature on grazing intensity and related indicators. Our analysis showed that grazing significantly affects CO_2_ emissions via changes in AGB, height, coverage, SM, and the concentrations of NH_4_^+^ and NO_3_^−^ in the soil (*p* < 0.05). More rainfall and less grazing result in greater CO_2_ emissions.

## Discussion

### Responses of the ecosystem CO_2_ fluxes to grazing intensity

Understanding the effects of grazing on ecosystem CO_2_ fluxes is important for predicting the effects of global climate change and human activities on C dynamics. Although related research has provided detailed comparisons of ecosystem CO_2_ fluxes under different grazing intensities^20, 26, 27^, most studies in grasslands are of short duration and therefore may not accurately capture the ecosystem CO_2_ emissions associated with grazing by animals. Our study provides the first observational data collected over a relatively long period for assessing the effects of grazing intensity on ecosystem CO_2_ fluxes in an Inner Mongolian meadow steppe ecosystem in China. This study enabled us to (1) directly test the effects of grazed vs. ungrazed conditions, (2) provide a more representative estimate of the yearly emissions rate during the growing and grazing season, and (3) allow for a more in-depth analysis of grazing treatment effects. The outcomes of this study do not support the hypothesis of higher CO_2_ fluxes under grazed native vegetation at higher stocking rates^28^. In our study, the grazed steppe functioned as a C source, and the peak CO_2_ fluxes during the growing season usually occurred after effective rainfall.

Our multi-level grazing intensity experiment presents robust evidence of the changes in ecosystem CO_2_ fluxes in the Hulunber *L. chinensis* meadow steppe in response to grazing intensity. Grazing decreased ecosystem CO_2_ emissions rates, and the ecosystem CO_2_ fluxes decreased with increasing grazing intensity. Significant negative linear relationships were found between the ecosystem CO_2_ fluxes and grazing intensity with an increasing grazing time. This pattern is consistent with the results of other grassland studies^29, 30^, but our results contradict reports suggesting that grazing increased the CO_2_ fluxes in a semi-arid mixed-grass prairie^20, 28^ and a shortgrass steppe in Colorado^31^. The differences may be attributed to the study site, climate, community type, size and composition of the C and nutrient pools^32^, quantities and composition of the soil microorganisms, physical and chemical properties of the soil^33^, and grazing intensity and history^34^. Firstly, grazing changes the plant community and soil environmental conditions, which determine the emissions of CO_2_ during the growing season. At higher stocking rates, the AGB, vegetation height, canopy cover and quantity of litter decreased at our sites^35^, and grazing resulted in lower plant cover and more bare soil, exposing a greater proportion of the soil surface to direct solar radiation, consequently increasing evaporative water loss. Plant height and surface litter decomposition are primarily moisture-dependent processes and are the factors that determine SM-holding capacity^35, 36^. Soil moisture declines more rapidly at grazed sites with little vegetation than at sites with denser and taller vegetation, and we also found a significant positive correlation between vegetation height and SM, which is therefore related to the soil CO_2_ flux. Secondly, grazing can affect the CO_2_ flux indirectly by removing live plant biomass, thereby decreasing the substrate available for soil biota^37^, or by altering plant height and canopy cover, which can affect the chemical composition of the input from the accumulated ground litter into the soil^26, 38, 39^, which in turn restricts CO_2_ production rates. Thirdly, with an increase in the grazing duration, the ecosystem CO_2_ flux began to be significantly affected by the heavy grazing, likely due to trampling, the deposition of dung, wallowing, and other physical activities. Long-term heavy grazing has been shown to significantly decrease the storage of soil C and N and cause grassland degradation^40^, which may significantly decrease the ecosystem CO_2_ flux.

### Effects of the main factors on CO_2_ fluxes with grazing intensity

Empirical relationships have been established between the observed CO_2_ fluxes and climate, soil, and plant factors. Precipitation and temperature are considered the most important factors determining the spatial variations in soil respiration^41^, and we found that the CO_2_ flux was significantly positively related to rainfall and negatively correlated with air temperature. Our analysis showed that rainfall, rather than air temperature, is the critical climatic factor determining ecosystem CO_2_ fluxes under the different grazing intensities. These results are consistent with observations from other arid ecosystems^29^. In a previous study that used rainfall manipulation shelters in the Konza Prairie, individual rain events were reported to increase the CO_2_ fluxes, whereas they tended to decrease with a prolonged dry period^42^. Our results indicate that the larger rain events were more efficient than the smaller rain events in stimulating ecosystem CO_2_ fluxes. Although several previous studies showed that warmer temperatures enhanced CO_2_ production in different soil types^43^, our study showed a negative correlation between CO_2_ production and air temperature, possibly due to global warming^44^.

Across all treatments and years, our results showed that the CO_2_ emissions rate was significantly positively correlated with SM, C/N ratio, SAP, soil NH_4_^+^-N, and soil NO_3_^−^N. This indicates that CO_2_ fluxes from semi-arid ecosystems are mostly limited by SM and inorganic N content, which is consistent with the findings of previous studies^21, 45–47^. It is well known that environmental factors, such as SM, influence soil biological activity and CO_2_ diffusion and therefore have pronounced influences on seasonal C exchange dynamics^48–50^. However, a negative relationship between soil respiration and SM was observed in an old-field grassland with a very high mean SM content^51^. This response may largely result from a reduction in the available oxygen for both microbial decomposition and autotrophic activities^48^. In our study, negative relationships were present between the ecosystem CO_2_ flux and TN and MBN, but our results do not agree well with the results of previous studies showing positive relationships between soil respiration and site traits such as soil C and total N contents^52^. These authors reported that the soils with higher total C and N contents typically emitted more CO_2_ than the grassland soils with lower total C and N contents. To specifically address environmental conditions in the Hulunber meadow steppe of Inner Mongolia, we also considered the effects of AGB, BGB, plant height, canopy cover and litter quantity on water capture, with denser and taller vegetation exerting a major influence on the potential water infiltration during plant growth periods. We found that the soil CO_2_ emissions rate was significantly positively correlated with AGB, plant cover, plant height and litter and was significantly negatively correlated with BGB across all sites. Hence, our results showed that grazing can also affect plant physiological processes and resource allocation between shoots and roots, thereby altering ecosystem CO_2_ emissions. These results are consistent with the results of previous studies^18^ in other ecosystems.

In our study, we showed that greater rainfall and lower temperatures lead to increased CO_2_ emissions and that low levels of CO_2_ emissions are closely related to MBN, MBC and BGB, but high levels of CO_2_ emissions are related to rainfall, AGB, height, coverage, SM, NH_4_^+^, and NO_3_^−^. The latter factors show significant positive correlations with CO_2_ fluxes, corroborating the findings of the correlation analysis. Therefore, the factors that affect CO_2_ fluxes include AGB, BGB^53^, SM^17^, canopy cover, community composition, and soil nutrient concentration^54^.

Additionally, CO_2_ fluxes may respond directly to variations in climatic factors, which may also indirectly affect fluxes by altering the response of the biota to environmental drivers^14^. Our findings support previous results showing that intra- and inter-year variations in rainfall and temperature can affect fluctuations in plant functional group composition and annual net primary productivity (ANPP) in semi-arid grasslands^53^. Overall, our analysis showed that grazing intensity significantly affects CO_2_ fluxes via changes in AGB, height, coverage, SM, and NH_4_^+^ and NO_3_^−^ concentrations in the soil and that greater rainfall and less grazing result in greater CO_2_ fluxes. Therefore, our study provides important information on the CO_2_ flux mechanisms, highlights the effects of different grassland grazing intensities on CO_2_ flux and the correlations between CO_2_ flux and environmental factors, and reveals key drivers in the C cycle in the plant community and soil environment that are mostly likely to affect CO_2_ emissions in Inner Mongolian meadow steppes.

## Conclusions

- Based on the CO_2_ flux, the Hulunber steppe ecosystem functioned as a C source during the growing and grazing season.
- The peak CO_2_ flux during the growing season usually occurred after an effective rainfall.
- Significant differences in CO_2_ fluxes were observed in response to differences in grazing intensity as well as with month and year.
- The effects of light and intermediate grazing remained comparatively constant with grazing year, whereas the effects of heavy grazing increased substantially with grazing duration.
- CO_2_ flux significantly decreased with increasing grazing intensity and duration.
- Our analysis showed that grazing intensity significantly affects the CO_2_ flux via changes in AGB, height, coverage, SM, and the concentrations of NH_4_^+^ and NO_3_^−^ in the soil and that greater rainfall and less grazing result in greater CO_2_ fluxes.
- Grazing and climate factors significantly affected ecosystem CO_2_ emissions either directly or indirectly via the modification of the plant community and soil environment.
- Our study provides important information to better evaluate the role of livestock grazing management in regulating GHG emissions.

## Materials and Methods

### Study area

**Figure 5:**
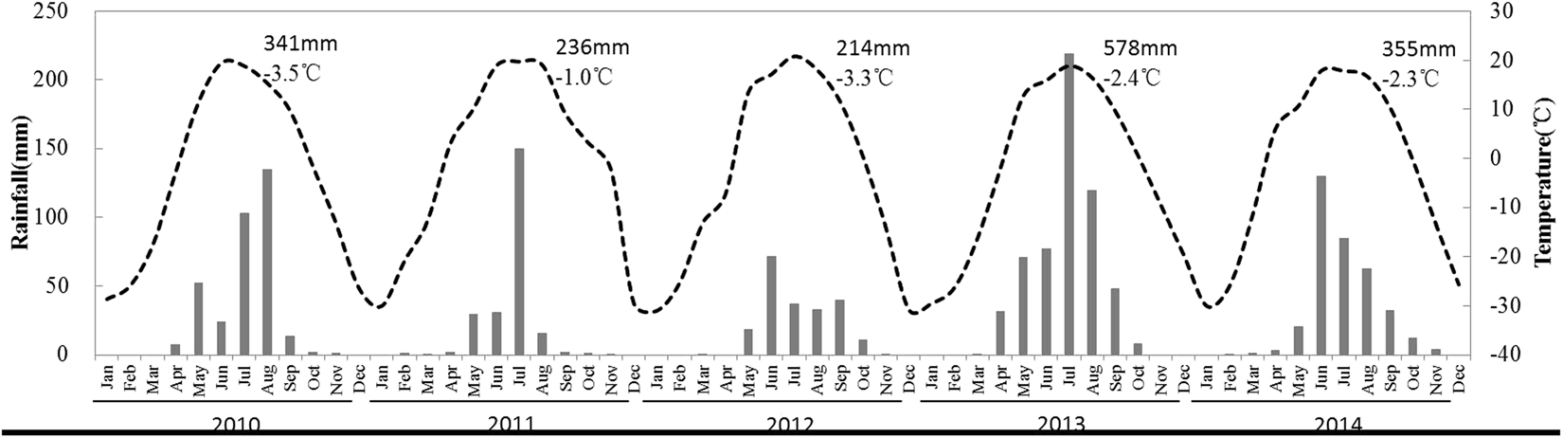
Monthly rainfall and temperature in 2010–2014 for the grazing experiment site. The values shown in each panel are the total annual rainfall and mean temperature.

This study was conducted at the Hulunber Grassland Ecosystem Observation and Research Station located at Xiertala farm in the centre of the Hulunber meadow steppe (N49°19′349′′, E 119°56′521′′) in the north-eastern region of Inner Mongolia, China. The elevation varies from 666 to 680 m. The climate is characterized as continental, temperate, and semi-arid, with an annual average of 110 frost-free days. The annual mean precipitation ranges from 350 to 400 mm, approximately 80% of which falls between July and September. The annual mean air temperature in this area is −5 to −2 °C, and the highest and lowest daily temperatures of 36.2 °C and −48.5 °C occur in January and July, respectively. Monthly average temperature and precipitation data from 2010–2014 for the study site are shown in Fig. 5. The vegetation is characterized as a typical Leymus chinensis and forbs meadow steppe. The dominant species are *L*. *chinensis*, *Scutellaria baicalensis*, *Carex pediformis*, *Galium verum*, *Bupleurum scorzonerifolium* and *Filifolium sibiricum*. The soil is characterized as a chernozem, or chestnut, soil.

### Experimental design

**Figure 6:**
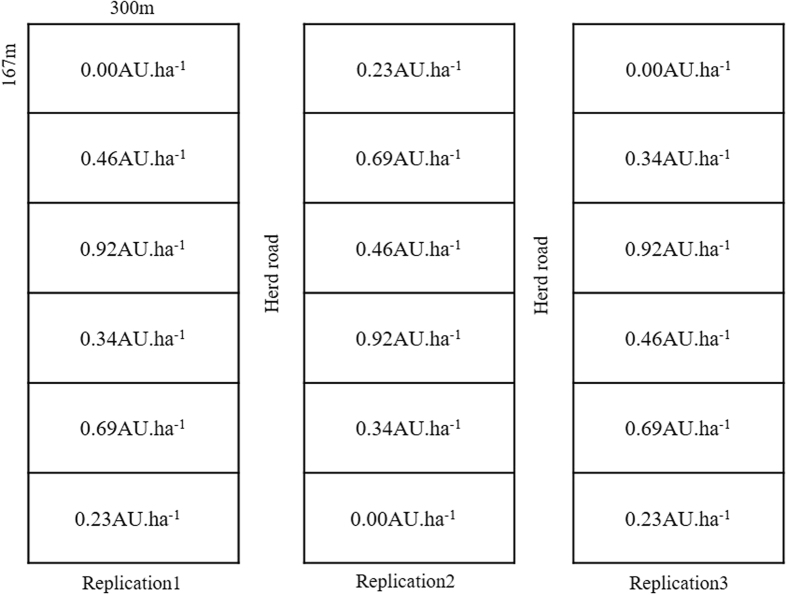
Experimental design and plot layout (0.00, 0.23, 0.34, 0.46, 0.69 and 0.92 AU ha^−1^, where 1 AU = 500 kg of adult cattle). The stocking rates were achieved using 0, 2, 3, 4, 6 or 8 young cattle (250–300 kg) per plot.

The grazing experiment was established in 2009 with six stocking rates (0.00, 0.23, 0.34, 0.46, 0.69, and 0.92 Animal Units ha^−1^, where 1 Animal Unit (AU) = 500 kg of adult cattle), with three replicates for each stocking rate and each replicate occupied a 5-ha paddock. Hence, in total, there were 18 plots randomly distributed over a total homogeneous area of 90 ha (Fig. 6). The stocking rates were achieved by using 0, 2, 3, 4, 6 or 8 young cattle (250–300 kg) per plot. Continuous grazing lasted for 120 days between June and October from 2009 to 2014. The grazing cattle were kept in the grazing plots day and night, and their drinking water was supplied from an outside water source. Before being fenced, the site was part of a larger area under long-term free-ranging cattle grazing. In the summer of 2008, baseline measurements were taken prior to the implementation of the field treatments using a 50-m transect in each plot to characterize the vegetation and soil traits^40^.

### Measurement of CO_2_ fluxes

CO_2_ fluxes were measured using the opaque static chamber method^55^. The static chamber system consisted of a stainless-steel frame (without a top and bottom, length × width × height = 50 cm × 50 cm × 10 cm) that was driven into the soil (installed prior to treatment initiation in August 2009) and a stainless-steel chamber (without a bottom, length × width × height = 50 cm × 50 cm × 50 cm) that was placed tightly in the base groove during the sampling period. The square box was inserted directly into the meadow soil to a depth of approximately 10 cm below the soil surface. The cover was placed on top during sampling times and removed afterwards. A fan 10 cm in diameter was installed in the top of each chamber to generate turbulence when the chamber was closed. The external surface of each chamber was covered with white plastic foam to minimize the effects of direct radiative heating during sampling. Three replicate chambers were randomly established in each plot and used for simultaneous measurements of the CO_2_ flux. The headspace in each chamber was sampled at intervals of 0, 10, 20 and 30 min after the chamber was closed. The gas was transferred immediately into a pre-evacuated 50 mL air bag using a 60-mL plastic syringe (Hede Inc., Dalian, China). The headspace CO_2_ concentrations were sampled twice per month during the growing season (June-October) in 2010 and four times per month during the growing season from 2011 to 2014. All measurements were taken between 9 and 11 a.m. The CO_2_ concentrations of the gas samples (stored in specific air bags) were analysed within one week using gas chromatography (Agilent 7890 A, Agilent Technologies Limited Co., USA). The CO_2_ flux was calculated according to Zhang *et al*.^56^.

### Measurements of auxiliary factors

#### Climate factors

Rainfall and temperature data were collected from an automatic meteorological station (Milos 520, Vaisala, Finland) at 30-min intervals.

#### Plant factors

Each month, five 1-m^2^ quadrats were randomly placed in each grazing plot during the growing season period from June to October in 2010, 2011, 2012, 2013 and 2014. Within each quadrat, the species composition and canopy height (cm) and cover (%) of each species were measured. A 50-cm × 50-cm point frame divided into a grid with 100 squares was used to measure coverage, and plant height was measured using a multipoint method with a ruler and averaged. The forage within the quadrat was cut and the AGB was oven dried for 48 h at 65 °C to constant weight. Litter was collected from the different grazing plots and weighed to the nearest 0.01 g with an electric balance. The BGB samples for all three replicates in each plot were collected in early August in 2010 to 2014. A soil pit was dug to a depth of 60 cm and the root mass in a 30-cm × 30-cm column was extracted from the depth ranges of 0–10, 10–20, 20–30, 30–40, 40–50, and 50–60 cm and washed through a 1-mm sieve. Fine roots or segments were retained on 0.25-mm sieves. The screened materials were further washed to separate the roots from the soil. All the roots were oven-dried at 80 °C for 12 h prior to weighing.

#### Soil factors

Each year, soil samples were taken from ten points per plot (to a depth of 10 cm) at the beginning of August in each year. The samples were combined to form a composite sample for each plot and stored at 4 °C in a refrigerator. One part was kept fresh for the measurement of soil NH_4_^+^ and NO_3_^−^ using a flow injection autoanalyser (FIAstar 5000 Analyzer, Foss Tecator, Denmark). The other part was used fresh for the measurement of MBC using fumigation extraction-capacity analysis and MBN and the fumigation extraction-indene three ketone colorimetric method. The remaining material was air-dried and ground for soil nutrient analysis^57^. All results are expressed on a dry weight basis. The SOC was determined using the dichromate oxidation method; the TN was determined using semi-micro Kjeldahl determination; the TP was determined using the molybdenum antimony resistance colorimetric method; the TK was determined using the NaOH molten flame photometer method; the SAN was determined by distillation; the SAP was determined using 0.5 mol/L sodium bicarbonate extraction; and SAK was determined by NH_4_OAc extraction and flame photometry^35^. Soil pH was measured using the electrode method; SBD was measured using the oven drying method; and SM was measured using the ring knife method. The soil parameters and vegetation factors over the different grazing intensities for the five years of the experiment are detailed in Yan *et al*.^35^.

### Calculations and statistical analyses

The major data analysis methods adopted in this study are variance analysis, correlation analysis and correspondence analysis (i.e., ANOVA, CORR, and CORRESP, respectively). The analyses were performed on the platform SAS9.30. The CO_2_ fluxes showed various changes during the growing and grazing period on both monthly and yearly scales. Thus, using the ANOVA test, three factors (year, month and grazing intensity) were adopted in the model. First, the data were analysed using variance analysis (significance level *p* < 0.05) followed by a Duncan multiple comparison test to compare the means. A significant difference (*p* < 0.05) is indicated by different letters, whereas no significant difference is indicated by the same letter. The Pearson correlation analysis was adopted to analyse the correlation of the CO_2_ fluxes with respect to other factors. Correspondence analysis, i.e., R-Q factor analysis, was used to directly obtain the result of analysis on the Q factor from the analysis of the R factor. The relationship between indexes and observations can be directly illustrated using an analytical graph of the relationship between the quantified indexes and the observations from the analytical table once the quantified indexes are combined with the observations.

## Electronic supplementary material

Supplementary Information
