# Supplementary material for: Response of ecosystem CO2 fluxes to grazing intensities – a five-year experiment in the Hulunber meadow steppe of China
Source: Sci Rep. 2017 Aug 25;7:9491. doi: 10.1038/s41598-017-09855-1 (PMC5573324; doi:10.1038/s41598-017-09855-1)
Supplement: Supplementary file 1 — Supplementary Information [file 41598_2017_9855_MOESM1_ESM.pdf]

Response of ecosystem CO_2 fluxes to grazing intensities – a five-year experiment in the Hulunber meadow steppe of China

R.R. Yan[a], H.J. Tang[a], S.H. Lv[b], D.Y. Jin[a], X.P. Xin[a*], B.R. Chen[a], B.H. Zhang[a], Y.C. Yan[a], X. Wang[a], Philip J. Murray[c], G.X. Yang[a], L.J. Xu[a], L.H. Li[d] and S. Zhao[e]

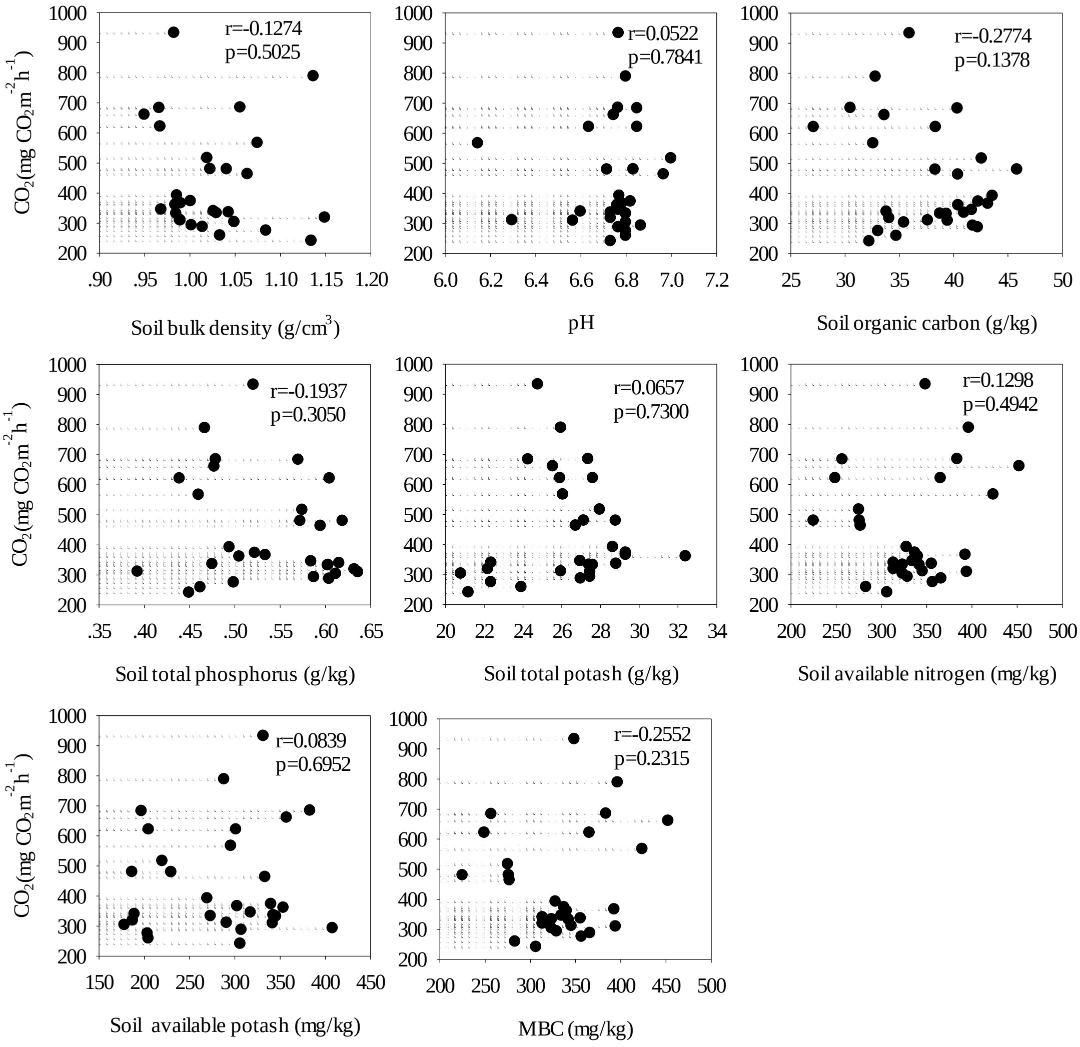

Supplementary Fig. 1. Relationships between the mean ecosystem CO_2 fluxes and soil other factors (soil bulk density, pH, soil organic carbon, soil total phosphorus, soil total potash , soil available nitrogen, soil available potash and MBC) from all plots across five years.
